# Multi-modal recommendation algorithm fusing visual and textual features

**DOI:** 10.1371/journal.pone.0287927

**Published:** 2023-06-29

**Authors:** Xuefeng Hu, Wenting Yu, Yun Wu, Yukang Chen

**Affiliations:** 1 The School of Electronics Engineering and Computer Science, Peking University, Beijing, China; 2 The State Key Laboratory of Public Big Data, Guizhou University, Guiyang, China; 3 The College of Computer Science and Technology, Guizhou University, Guiyang, China; University of Sindh, PAKISTAN

## Abstract

In recommender systems, the lack of interaction data between users and items tends to lead to the problem of data sparsity and cold starts. Recently, the interest modeling frameworks incorporating multi-modal features are widely used in recommendation algorithms. These algorithms use image features and text features to extend the available information, which alleviate the data sparsity problem effectively, but they also have some limitations. On the one hand, multi-modal features of user interaction sequences are not considered in the interest modeling process. On the other hand, the aggregation of multi-modal features often employs simple aggregators, such as sums and concatenation, which do not distinguish the importance of different feature interactions. In this paper, to tackle this, we propose the FVTF (Fusing Visual and Textual Features) algorithm. First, we design a user history visual preference extraction module based on the Query-Key-Value attention to model users’ historical interests by using of visual features. Second, we design a feature fusion and interaction module based on the multi-head bit-wise attention to adaptively mine important feature combinations and update the higher-order attention fusion representation of features. We conduct experiments on the Movielens-1M dataset, and the experiments show that FVTF achieved the best performance compared with the benchmark recommendation algorithms.

## Introduction

With the rapid development of Internet applications, service delivery platforms such as e-commerce, advertising, and movie websites generate a large amount of data every day, which brings us the problem of information overload, making it difficult for users to choose among thousands of items and find the content they want. Recommendation systems emerge to solve this problem by analyzing users’ historical behaviors and digging out their preference information to personalize and recommend items for them. On the one hand, it brings great business value to the company, and on the other hand, it improves user satisfaction and makes it easier for users to find the content they want.

Click-through rate prediction (CTR) [1] is a common method in recommender systems, which predicts users’ click-through rates on candidate items by mining their interests from their historical behaviors, and ranks the candidate items to recommend items that are more likely to be of interest and clicked by the users. And feature engineering is crucial to the accuracy of click-through prediction. For example, it is reasonable to recommend cosmetics to a woman, where <gender = female, product category = cosmetics>is a very useful set of second-order features. Another example is that it is reasonable to recommend a game console to a 9-year-old boy, where <gender = male, age = 9, product category = game console>is an important set of third-order feature combinations, and these important feature combinations will bring a great improvement to the prediction performance. Traditional CTR prediction mainly relies on manually crafted features and uses shallow models for prediction, such as Logistic Regression (LR) [2–4]. However, manual feature engineering greatly relies on domain experts and makes it difficult to find all useful combinations of features. In recent years, with the rise of deep learning, many deep learning-based CTR prediction algorithms [5–8] have overwhelmingly outperformed traditional algorithms. Such algorithms automate feature engineering using deep learning algorithms, which not only replace traditional complex manual feature engineering but also can tap into deeper feature information, i.e., higher-order feature combinations. Yet, all such algorithms inevitably need to cope with data sparsity and cold start problems. For example, new users or new items have few records, resulting in parameters that cannot be adequately trained. Therefore, it is crucial for recommendation algorithms to exploit the limited records to mine users’ preferences and recommend items more accurately.

To solve the problem of sparse data and cold start in recommender systems, in recent years some studies [9–11] have used multi-modal techniques in recommender systems to extend the user and item features. Among them, images are very important features, which are widely present in item descriptions and have a decisive role in predicting whether a user will click on an item or not. For example, when shopping on e-commerce sites, users browse products through search or personalized recommendations, and each product is usually presented to the user by an image of the product and some text describing the product, and when the user is interested in a product, he or she clicks on the image to see the details. Images can provide intrinsic visual descriptions that are very intuitive and influence people’s decisions. When shopping online, the user’s eye always catches the image of the product, rather than reading the textual description first. Moreover, users will only decide to like or dislike a garment after seeing an image of it. Since images of items are objects that users interact with directly, these images can provide visual information about users’ preferences, so incorporating image features into the recommendation algorithm to complement the text features will have a positive impact on the accuracy of the recommendation results.

In recent years, some researchers have added image features to the models, but they generally have the following problems: (1) First, only the image features of the target items are considered in the model design, failing to consider the influence of the image features of the user’s historical clicked items on the user’s visual preferences. (2) In feature fusion and interaction, the feature vectors are usually just joined together and input to MLP(Multilayer Perceptron) for implicit feature interaction, which ignores the importance of feature interaction and does not guarantee its effectiveness.

To address the above issues, in this paper, we propose a multi-modal recommendation algorithm (FVTF) that incorporates visual and textual features. First, we note that users have different attention for different visual features of target items. Traditional methods use simple operations such as pooling or concatenation to uniformly aggregate the representation vectors of image sequences, making the learned visual preference representations lack of targeting. In order to explicitly mine users’ visual preferences, we design a user visual preference extraction module based on the Query-Key-Value attention mechanism. The module learns different user visual attention based on the visual feature of the target item, which adaptively obtains the visual preference representation of user. Meanwhile, in the feature fusion and interaction approach, we design a feature fusion and interaction module based on a multi-head bit-wise attention mechanism to mine important feature combinations and extract higher-order attention fusion representations of visual features and text features.

To summarize, we make several noteworthy contributions in this paper.

- We design a module for user visual preference extraction based on the Query-Key-Value attention mechanism. The module takes image sequence data and visual features of target items as input, then adaptively learns users’ visual preference according to target item.
- We design a new feature fusion and interaction module, which mines important feature combinations using a multi-head bit-wise attention mechanism, and updates the higher-order attention fusion representation of features according to the importance of the feature combinations, fully preserving and utilizing the information of features from low to high order.
- We conducted experiments on the Movielens-1M dataset. The experimental results show that FVTF achieves the best performance compared to the state-of-the-art algorithm.

## Related work

### Single-modal recommendation algorithms

Early CTR prediction algorithms relied on well-designed statistical features, such as LR, Lightgbm, XgbBoost, and other algorithms. Such algorithms are unable to learn complex feature intersections and require a lot of manual feature engineering work, which makes feature engineering extremely difficult as the number of feature dimensions continues to increase. With the development of deep learning, the use of deep neural networks for building CTR prediction algorithms has greatly liberated manual feature engineering and can be used to extract complex higher-order feature interactions and improve the performance of the algorithms. Wide&Deep [5] and DeepFM [6] combine the higher-order interaction information extracted by deep neural networks with the first- and second-order interaction information extracted by LR and FM to combine the feature of low-order interaction information with higher-order interaction information for prediction, making the algorithm both memorization and generalization capabilities. algorithms such as AFM [12] and AutoInt [13] introduce attention mechanisms to distinguish the importance of feature interactions and better focus on the important feature interactions. To improve the attention mechanism and explicitly algorithm higher-order feature interactions to address the problem of uncontrollable neural network interactions, HoAFM [14] and EHAFM [15] used bit-wise attention mechanisms and designed explicit higher-order interaction algorithms, which significantly improved the algorithm performance and efficiency. However, the above algorithms only consider the features of text-modal, which can easily lead to the problems of data sparsity and cold start when there are few users and item behaviors, affecting the algorithm accuracy.

### Multi-modal recommendation algorithms

To solve the problem of sparse data and cold start, image features with visual semantic information can be complemented with textual information to bring better generalization capability to the algorithm. The emergence of deep learning networks facilitates the extraction of multi-modal features. For example, Chen et al. [16] proposed ACF model based on attention mechanism and Wei et al. [17] proposed GRCN model based on graph convolutional network to explore the problem of using deep networks to learn implicit feedback in item and multi-modal feature interaction. Wei et al. [18] designed Hierarchical User Intent Graph Network to learn multi-modal features from users’ co-interacted patterns to learn multi-level user intent,so as to obtain high-quality representations of users and items and further enhance the recommendation performance.

In recent years, research on image representation has achieved remarkable results, and learning high-wise semantic features by deep learning algorithms [19, 20] is effective in a large number of tasks. In some previous works, attempts have been made to introduce image features into CTR prediction algorithms by extracting image feature representations using CNN models with pre-trained parameters and combining them with other features for click-through prediction. Chen et al. [21] proposed a DEEPCTR algorithm to train CNNs in an end-to-end manner, fuse them with text features, and input them to MLP for CTR prediction. Ge et al. [22] argue that images of the target item describe the visual characteristics of the advertisement, while images of the user’s historical behavior reveal the user’s visual preferences, and that combining this visual information yields better performance than using either of them alone. The CMBF algorithm proposed by Chen et al. [23] uses a Multihead-Self-Attention mechanism [24] to separately image features and text features learning, followed by cross-fusion of features from both modalities.

However, existing multi-modal recommendation algorithms usually use stitching to treat the extracted multi-modal features as a whole when fusing multi-modal features, ignoring the variability of user preferences in feature interactions of multiple modal features. For example, the MMGCN [25] and MGAT [26], which learns the single-modal user preferences and concatenates them to represent the multi-modal user preference on the micro-video. To tackle this problem, Wang et al. [27] design a multi-modal representation learning module to explicitly model the user’s attentions over different modalities and inductively learn the multi-modal user preference. Chen et al. [28] propose the Edge-wise mOdulation (EGO) fusion operation, which distills edge-wise multi-modal information and learns to modulate each unimodal node under the supervision of other modalities. It breaks isolated single-modal propagations and allows information to be propagated between modalities.

## Our proposed method

### Overview

**Fig 1:**
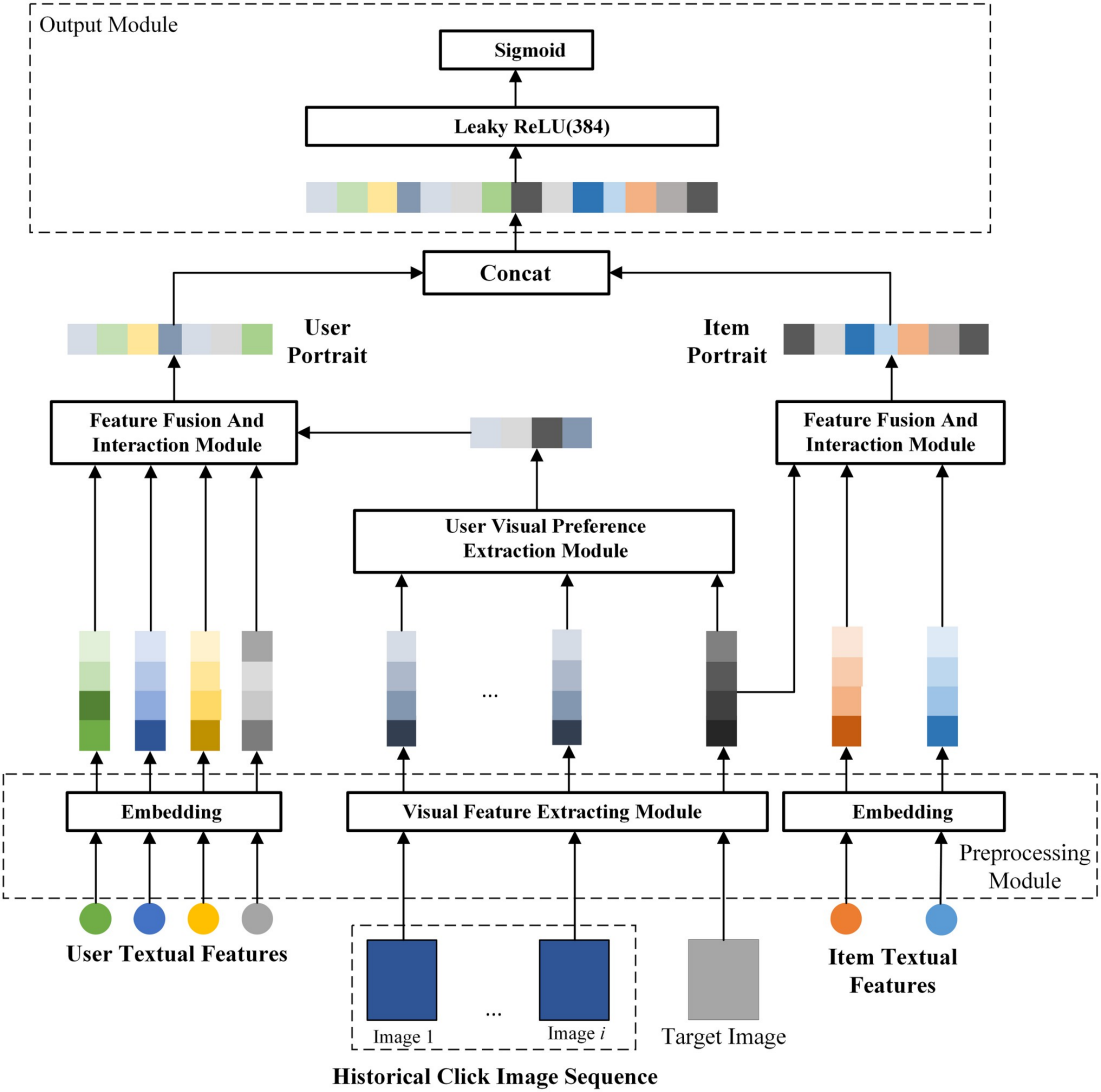
FVTF overall architecture.

FVTF algorithm is mainly divided into four parts: pre-processing module, user visual preference extraction module, feature fusion and interaction module, and output module, as shown in Fig 1.

First, in the feature preprocessing part, we use different methods to embed and encode the text features and image features of users and items. Then, the image embedding vector from the user’s historical behavior, and the target image’s embedding vector are fed into the user visual preference extraction module to obtain the user visual preference feature vector. Afterwards, the user visual preference feature vector and the target image embedding vector are fed into the feature fusion and interaction module to fuse and interact with the user-side and item-side text vectors respectively to obtain the user portrait and item portrait. Finally, in the output layer, the user portrait and the item portrait are joined together and input into a single-layer neural network to predict the user’s click rate by the sigmoid activation function.

### Pre-processing module

To extract the information of text features and image features, we design different methods to preprocess text features and image features in the preprocessing module.

For text features, to alleviate the detrimental effects of the high dimensionality and sparsity of the feature vector on training, we use the Embedding technique [29–31] for embedding dimensionality reduction of numerical features, single-valued classification features, and multi-valued classification features:

$$\begin{array}{c} t_{i}=\{\begin{array}{cc} w_{i}x_{i}, & \;\text{for}\;\text{numerical}\;\text{features}\\ W_{i}x_{i}, & \;\text{for}\;\text{single}\;-\;\text{value}\;\text{classification}\;\text{features}\\ \frac{1}{Q}W_{i}x_{i}, & \;\text{for}\;\text{multi}\;-\;\text{value}\;\text{classification}\;\text{features}\; \end{array} \end{array}$$ (1)

where *x*_*i*_ is the element in the text feature vector, *t*_*i*_ represents the reduced-dimensional embedding vector, *w*_*i*_ represents the Embedding Mapping Vector in the case of the numerical feature, *W*_*i*_ represents the Embedding Mapping Matrix in the case of the classification feature, and *Q* represents the number of all potential values if *x*_*i*_ is a multi-value feature vector.

Using the Embedding technique, we represent the set of text feature vectors *t*^*u*^ of user *u* with the set of text feature vectors *t*^*v*^ of item *v* as

$$\begin{array}{c} t^{u}=\{t_{1}^{u},t_{2}^{u},\ldots ,t_{m}^{u}\},t^{v}=\{t_{1}^{u},t_{2}^{v},\ldots ,t_{n}^{v}\} \end{array}$$ (2)

For image features, to extract the rich semantic information in them, in this algorithm we utilize an image feature extraction network based on EfficientNetB0 [32] for feature extraction. Thus, The set of feature vectors of historical click images of user *u* can be expressed as:

$$\begin{array}{c} I^{u}=\{I_{1}^{u},I_{2}^{u},\ldots ,I_{k}^{u}\} \end{array}$$ (3)

The image feature vector of the target item *v* can be represented as *I*^*v*^.

### User visual preference extraction module

**Fig 2:**
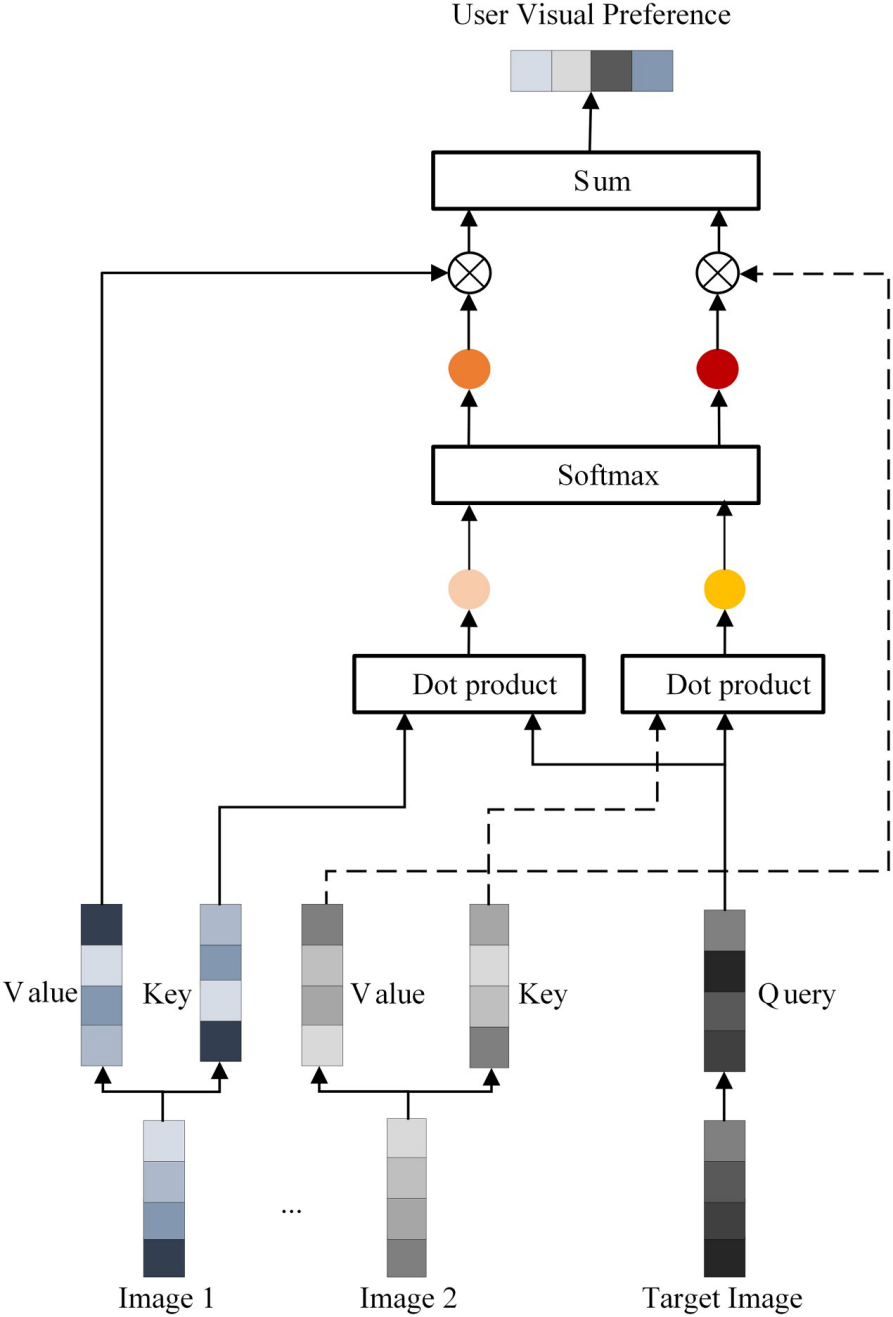
User visual preference extraction module.

The display image of an item often contains some elements about the content of the item, so the user’s visual preferences are embedded in the image of the item that the user has historically clicked on. To personalize the modeling of user visual preference features, we design a user visual preference extraction module, which uses the Query-Key-Value attention mechanism to mine the correlation between user history clicked images and target item images, and use it to pool the image features of user history clicked list to extract user visual preferences, as shown in Fig 2. Specifically, The correlation $a_{i}^{v,u}$ between the image features of the target item *v* and the image features of the *i*-th action of user *u* can be expressed as:

$$\begin{array}{c} a_{i}^{v,u}=\frac{\text{exp}(\psi (I^{v},I_{i}^{u}))}{\sum_{j=1}^{k}\text{exp}(\psi (I^{v},I_{j}^{u}))} \end{array}$$ (4)

$$\begin{array}{c} \psi (I^{v},I_{i}^{u})=<W_{\text{Query}\;}I^{v},W_{\text{Key}\;}I_{i}^{u}> \end{array}$$ (5)

Where *ψ*(*a*, *b*) denotes the similarity between vector a and vector b. It can be calculated using neural networks, vector inner product, and other methods. In this paper, we use vector inner product to calculate the similarity because of its simplicity and effectiveness.$W_{\text{Query}\;},W_{\text{Key}\;}\in R^{d^{{}^{\prime }}\times d}$ are the query projection matrix and the key projection matrix, respectively, which map the original embedding space **R**^*d*^ of the feature vector to a new space $R^{d^{{}^{\prime }}}$, where *d*′ needs to be consistent with the embedding dimension of the text features to ensure that the text feature vector and the image feature vector can be fused and interacted in the later part.

Next, we obtain the visual preference feature vector *I*^*v*,*u*^ of user *u* based on the correlation between the image features of target item *v* and the image features of all actions of user *u*, as shown below.

$$\begin{array}{c} I^{v,u}=\sum_{i=1}^{k}a_{i}^{v,u}(W_{\text{Value}\;}I_{i}^{u}) \end{array}$$ (6)

where $W_{\text{Value}}\in R^{d^{{}^{\prime }}\times d}$ is the value projection matrix.

### Feature fusion and interaction module

Currently, most recommendation algorithms use feature vectors of different modalities directly stitched together and input to MLP for implicit feature interaction when fusing and interacting with features of different modalities. And this approach does not distinguish the importance of different feature combinations. In addition, the effectiveness of feature interaction cannot be guaranteed due to the black-box nature of neural networks. In this paper, a new feature fusion and interaction module is designed, which consists of multiple feature fusion and interaction layers, and in each layer, the importance of each feature interaction is pooled according to the importance of each feature interaction using a multi-head bit-wise attention mechanism to increase the impact of important feature interactions and reduce the interference of useless feature interactions. And the feature higher-order fusion representation is obtained. In previous work, the effectiveness of using this method to explicitly model feature interactions is verified, which possesses better performance than vector-wise attention mechanisms such as Soft Attention [33] and multi-head Self-Attention [24].

**Fig 3:**
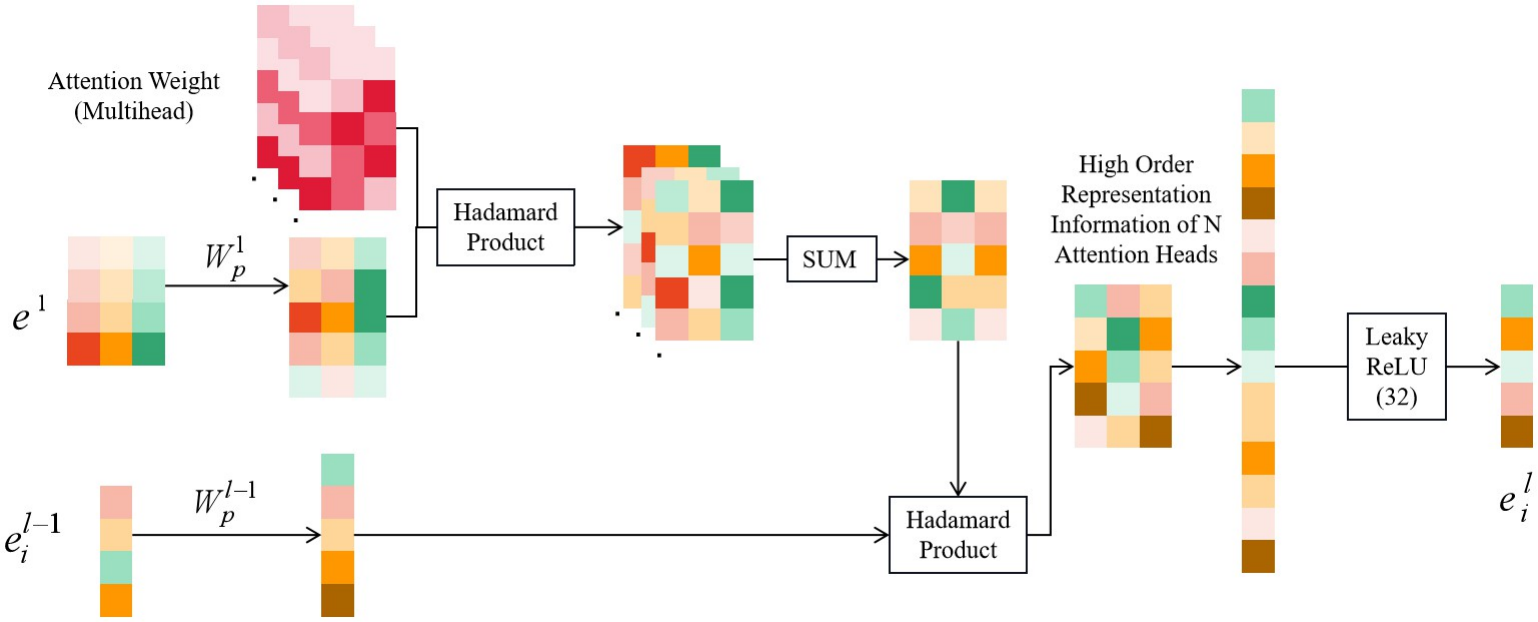
Schematic diagram of feature fusion and interaction module.

The schematic diagram of the feature fusion and interaction layer computation method is shown in Fig 3. In multimodal feature fusion, the new text feature representation contains the information of image features, and the new image feature representation contains the information of text features. And by increasing the number of feature fusion and interaction layers, the higher-order representation of text features as well as image features can be obtained, and the valuable deep information in the features can be mined.

Let the dimensionality of all feature vectors of the input module be *d*′, and in order to enhance the expressiveness of the feature vectors, the model uses a trainable projection weight matrix to map the feature vectors to a high-dimensional space and expand the feature vector dimensionality, which is calculated as shown in the following equation.

$$\begin{array}{c} E_{i}^{l}=W_{p}^{l}e_{i}^{l} \end{array}$$ (7)

Where: $e_{i}^{l}\in R^{D}$ denotes the *l*-th order feature vector of feature *i*, $e_{i}^{1}\in R^{D}$ denotes the first-order feature vector of feature *i*, and $E_{i}^{l}$ is the l-order feature enhancement representation of feature i. $W_{p}^{l}$ is the l-order projection matrix, which is used to project the feature vector of dimension *d*′ into the *D* dimension.

Then, the importance of feature interactions is mined using the bit-wise attention mechanism, and pooled according to the importance of each dimension, and fused to form a higher-order representation of the features. The *l*-th order representation of feature *i* can be formulated as:

$$\begin{array}{c} e_{i}^{l}=\sum_{j=1}^{M}(w_{i,j}^{l-1}⊙E_{i}^{l-1}⊙E_{j}^{1})=E_{i}^{l-1}⊙\sum_{j=1}^{M}(w_{i,j}^{l-1}⊙E_{j}^{1}) \end{array}$$ (8)

where: ⊙ denotes the Hadamard product of two vectors,(*a*_1_, *b*_1_, *c*_1_) ⊙ (*a*_2_, *b*_2_, *c*_2_) = (*a*_1_*a*_2_, *b*_1_*b*_2_, *c*_1_*c*_2_), and we use the Hadamard product of two feature vectors to denote the interaction of two vectors;$w_{i,j}^{l-1}\in R^{D}$ denotes the (*l* − 1)-th order of feature *i* denotes the attention weight vector interacting with feature *j*; M is the number of feature vectors.

To avoid overfitting, we create multiple attention heads that do not share parameters for feature interaction importance learning, learn feature interaction importance in different representation subspaces, respectively, and finally integrate the output of multiple attention heads, which is formulated as follows:

$$\begin{array}{c} \;\text{head}{\;}_{i,h}^{l}=E_{i}^{l-1}⊙\sum_{j=1}^{M}(w_{i,j}^{l-1,h}⊙E_{j}^{1}) \end{array}$$ (9)

$$\begin{array}{c} \;\text{head}{\;}_{i}^{l}=\;\text{head}{\;}_{i,1}^{l}⊕\;\text{head}{\;}_{i,2}^{l}⊕\cdots ⊕\;\text{head}{\;}_{i,H}^{l} \end{array}$$ (10)

$$\begin{array}{c} \;\text{head}{\;}^{l}=[\;\text{head}{\;}_{1}^{l},\;\text{head}{\;}_{2}^{l},\ldots ,\;\text{head}{\;}_{M}^{l}] \end{array}$$ (11)

where: ⊕ is the jointing operation, H is the total number of attention heads, and the weight parameters of each attention head are independent of each other.

To combine the information from multiple attention heads, we perform a linear transformation to obtain a vector *e*^*l*^ of *l*-th order representations of all features:

$$\begin{array}{c} e^{l}=\mu (W_{mh}^{l}\;\text{head}{\;}^{l}) \end{array}$$ (12)

Where: *μ*(•) is the activation function, and the LeakyReLU activation function is chosen in this paper; $W_{mh}^{l}\in R^{D\times DH}$ is the projection vector, which transforms the representation vector from DH dimension to D dimension.

By stacking multiple feature fusion and interaction layers, the 1-st to *l*-th order representation tensor *Z* = [*e*^1^, *e*^2^, …, *e*^1+1^] ∈ **R**^*l*×*M*×*D*^ of the features is obtained. finally, the tensor *Z* is flattened to obtain the output *p* of the feature fusion and interaction module, and for the convenience of representation, the feature fusion and interaction module is expressed functionally as follows:

$$\begin{array}{c} p=\;\text{Interaction}\;(e^{1}) \end{array}$$ (13)

The features of the input module are divided into user-side features and item-side features. First, all the text feature vectors of user-side and user visual preference feature vectors are formed into a user features set *e*^*u*^, as shown in the following:

$$\begin{array}{c} e^{u}=\{t_{1}^{u},t_{2}^{u},\ldots ,t_{m}^{u},I_{v,u}\}=\{e_{1}^{u},e_{2}^{u},\ldots ,e_{m+1}^{u}\} \end{array}$$ (14)

Then, the user feature set will be input to the feature fusion and interaction module to obtain the user portrait feature *p*_*u*_.

$$\begin{array}{c} p_{u}=\;\text{Interaction}\;(e^{u}) \end{array}$$ (15)

For item-side features, the target item image feature vector is first mapped to an embedding representation space of the same dimension as the text feature vector, as shown in the following equation:

$$\begin{array}{c} I^{v^{\prime }}=W^{v}I^{v} \end{array}$$ (16)

where *W*^*v*^ ∈ **R**^*d*′×*d*^, d is the text feature vector dimension and *d*′ is the target item image feature vector dimension.

The set of item features is formed by combining all text feature vectors with the target item image feature vectors *e*^*v*^, and will be fed into the feature fusion and interaction module to obtain the item portrait features *p*_*v*_, as fowllows:

$$\begin{array}{c} e^{v}=\{t_{1}^{v},t_{2}^{v},\ldots ,t_{n}^{v},I^{v^{\prime }}\}=\{e_{1}^{v},e_{2}^{v},\ldots ,e_{n+1}^{v}\} \end{array}$$ (17)

$$\begin{array}{c} p_{v}=\;\text{Interaction}\;(e^{v}) \end{array}$$ (18)

### Output layer

In the output layer, we splice the user portrait feature *p*_*u*_ with the item portrait feature *p*_*v*_ and learn the relationship between *p*_*u*_ and *p*_*v*_ through a feed forward neural network to predict the user’s click rate on the target item, which is formulated as follows:

$$\begin{array}{c} a^{0}=p_{u}⊕p_{v} \end{array}$$ (19)

$$\begin{array}{c} a^{h+1}=\;\text{LeakyReLU}\;(W^{h+1}a^{h}+b^{h+1}) \end{array}$$ (20)

$$\begin{array}{c} \hat{y}=\text{Sigmoid}(W^{l+1}a^{l+1}+b^{l+1}) \end{array}$$ (21)

where: ⊕ denotes two vectors for jointing operation; *l* denotes the number of layers of the feedforward neural network; *a*^*h*^, *W*^*h*^, *b*^*h*^ denote the output of the *h*-th layer, the weight matrix, and the bias vector, respectively.

Finally, the output of the last layer is passed through the Sigmoid activation function for CTR prediction.

Click-through rate prediction is a dichotomous task (click, unclick), so the loss function of this algorithm uses logarithmic loss, and in addition, we add an L2 regularization term to the algorithm to prevent overfitting problems:

$$\begin{array}{c} L=-\frac{1}{N}\sum_{j=1}^{N}(y_{j}\text{log}({\hat{y}}_{j})+(1-y_{j})\text{log}(1-{\hat{y}}_{j})+\lambda {\left\|\Phi \right\|}_{2}) \end{array}$$ (22)

Where: *N* is the total number of training samples, *y* is the sample true label (1 or 0),$\hat{y}$ is the predicted value of the sample, *ϕ* is the set of trainable parameters of the algorithm, and *λ* is the manually adjustable L2 regularization factor.

## Experiments

### Dataset

This experiment uses the Movielens-1M [34] dataset, a set of movie rating data collected by GroupLens Research from MovieLens users from the late 1990s to the early 2000s, containing about 4000 movies from about 6000 users with about 1 million user rating records. It contains data on users’ ratings of movies, users’ gender, age, occupation, movie genre, and movie era. The dataset lacks movie image information, but provides the imdbID of each movie. based on the imdbID, we can look up the corresponding movie information on the IMDB website, which contains movie posters, and we use crawling techniques to get the corresponding posters of each movie.

For data preprocessing: for text features, the null values are filled with -1. In the user history behavior sequence construction, the five click records before the user’s behavior with the target item according to the timestamp are used as the user history behavior sequence to capture the user’s recent preferences. Since some movies failed to obtain posters, we delete this part of user behavior data. We preprocessed the image information and changed the size to 64×64×3, and used the pre-trained EfficientNetB0 model on ImageNet (ISVRC2012) as the image feature extraction module to extract the image features of movie posters. For the sample labels, the samples with user ratings greater than 3 in the Movielens-1M dataset are regarded as positive samples (label = 1) and those less than 3 are regarded as negative samples (label = 0), and the neutral samples with a rating of 3 are removed. The data set was randomly disrupted and partitioned into the training set, validation set, and test set according to 8:1:1, and the information of the pre-processed data set is shown in Table 1.

**Table 1:** Dataset attributes.

	Attributes	Dimension
*User*	Gender	2
	Occupation	21
	Age	61
	Zip-code	795
*Movie*	Type	19
	Year	37
	Image	128
*Others*	Timestamps	1

### Benchmark algorithms

We compare FVTF with the following algorithms, which can be divided into classical algorithms used before the emergence of deep networks, single-modal recommendation algorithms based on deep learning, and deep learning recommendation algorithms that incorporate multi-modal features.

(1)Classic algorithms before the advent of deep neural networks

- LR [2]: logistic regression, an algorithm that uses only first-order features for prediction.
- FM [4]: Factorization Machines, which represents second-order feature interactions using the dot product of two feature vectors.

(2)Single-modal recommendation algorithms based on deep learning.

- Wide&Deep [5]: combining logistic regression for extracting first-order features as the Wide part and deep neural network for extracting higher-order features as the Deep part, making the model both memorization and generalization capabilities.
- DeepFM [6]: a model that combines a factorization machine and a deep neural network for extracting second-order interaction features and higher-order interaction features, respectively.
- xDeepFM [7]: compression of the feature interaction tensor using convolutional neural networks, capable of explicitly modeling higher-order interactions at the vector wise.
- AutoInt [13]: updates the feature interaction representation using a multi-head self-attentive mechanism.
- HoAFM [14]: explicitly models feature higher-order interactions based on a bit-wise attention mechanism.
- EHAFM [15]: expands the feature representation dimension based on HoAFM and adds multiple attention heads to enrich the feature representation information.

(3) Deep learning recommendation algorithms incorporating multi-modal features

- MLFM [10]: is a multi-modal post-fusion classification method based on text and images. It uses machine learning models to extract text and image features, learns specific classifiers for each modality, and then learns fusion strategies from the results of each modality classifier.
- VBPR [11]: incorporates visual information into the recommendation model, which is a significant improvement over the matrix decomposition model that relies on the hidden vectors of users and items.
- DICM [22]: this method extracts user visual preference features using the item image features of the user’s historical behavior and the image features of the target item, stitches them with the user’s ID features and the image features of the target item, and inputs them into a deep neural network for click-through rate prediction.
- CMBF [23]: They proposed a new cross-modal fusion method based on the Multihead-Self-Attention mechanism for the complete fusion of multi-modal features to learn cross-information between different modalities.

(3) Deep learning recommendation algorithms incorporating multi-modal features

- MLFM [10]: is a multi-modal post-fusion classification method based on text and images. It uses machine learning models to extract text and image features, learns specific classifiers for each modality, and then learns fusion strategies from the results of each modality classifier.
- VBPR [11]: incorporates visual information into the recommendation model, which is a significant improvement over the matrix decomposition model that relies on the hidden vectors of users and items.
- DICM [22]: this method extracts user visual preference features using the item image features of the user’s historical behavior and the image features of the target item, stitches them with the user’s ID features and the image features of the target item, and inputs them into a deep neural network for click-through rate prediction.
- CMBF [23]: They proposed a new cross-modal fusion method based on the Multihead-Self-Attention mechanism for the complete fusion of multi-modal features to learn cross-information between different modalities.

### Experimental setup

The algorithm is implemented using Tensorflow 2.0. In the parameter settings, the Embedding dimension of text features is set to 16 and the Embedding dimension of image features is set to 128 in the preprocessing module; In the feature fusion and interaction module, the number of layers of feature interaction layer is set to 4, the number of attention heads is set to 2, and the feature fusion dimension is set to 32. In the output layer, a neural network with 384 neurons is used for learning; and the L2 Regularization factor is set to 0.0001. The above parameters are the optimal parameters obtained from the experiments.

In the training strategy, Adam [35] is used as the optimizer, and the batchsize is set to 1024. 30 epochs are trained with a Learning Rate of 0.01, and then 10 epochs are trained with a lower Learning Rate of 0.001, and an early stopping strategy is used to prevent overfitting. As shown in Table 2.

**Table 2:** Experimental setup.

Parameter	Value
*Optimizer*	Adam
*BatchSize*	1024
*LearningRate*	0.0001
*TextEmbeddingdimension*	16
*ImageEmbeddingdimension*	128
*FeatureFusionDimension*	32
*Numberoflayersofinteraction*	4
*Numberofattentionheads*	2
*MLPPart*	1 Layer,384 Hidden Neurons
*L*2*Regularizationfactor*	0.0001

### Evaluation metrics

The experiments in this paper use AUC and Logloss as evaluation metrics.

AUC (Area under the curve): AUC is the area under the ROC curve, which measures the probability that the positive cases are ranked in front of the negative cases in the algorithm prediction, and the closer its value is to 1, the better the dichotomous classification performance and the indicator is insensitive to whether the sample categories are balanced or not, and can still make reasonable predictions in the case of sample imbalance, which is the most important evaluation index in the click-through prediction task.

Logloss: Logloss is also a widely used evaluation criterion in dichotomous classification tasks, which can measure the degree of fit between the predicted CTR and the actual CTR, the lower the Logloss the better the fit ability.

### Experimental results and analysis

#### Performance comparison

We compare the performance of FVTF with twelve benchmark algorithms. The results are shown in Table 3. We summarize the experimental analysis as follows.

**Table 3:** Performance comparison of different algorithms.

Alogorithm	AUC	Logloss
LR	0.7775	0.5441
FM	0.7777	0.5176
Widedeep	0.7920	0.4683
DeepFM	0.7809	0.4351
xDeepFM	0.8324	0.3914
AutoInt	0.8340	0.3953
HoAFM	0.8349	0.3922
EHAFM	0.8429	0.3805
VBPR	0.8419	0.3700
MLFM	0.8489	0.3731
DICM	0.8655	0.3665
CMBF	0.8836	0.3302
**FVTF**	**0.8945**	**0.3176**

Compared with traditional classical algorithms such as LR and FM, the algorithms based on deep learning achieve a great performance improvement. This indicates that deep networks have a stronger ability to acquire interaction features compared to manual feature engineering. Meanwhile, it can be seen that multi-modal recommendation algorithms have better performance compared to single-modal. This indicates that introducing features of different modalities into the algorithmic framework effectively extends the available information to extract more information about interaction features, which positively contributes to improving the algorithm performance.

Our proposed FVTF achieves the best performance. On the one hand, compared with classical algorithms and single-modal recommendation algorithms based on deep learning, This is because FVTF utilizes multimodal information to alleviate data sparsity, incorporates item image features to complement the features, and captures user preferences from multiple modal features to provide more accurate recommendations. On the other hand, compared with multi-modal algorithms, FVTF also has performance improvement. Particularly, compared with DICM, FVTF improves by 3.3% in terms of AUC. This confirm that the bit-wise attention based adaptive feature fusion approach is more effective and flexible than the simple fusion (i.e., concatenation) to obtain interaction features.

#### Training time comparison

**Fig 4:**
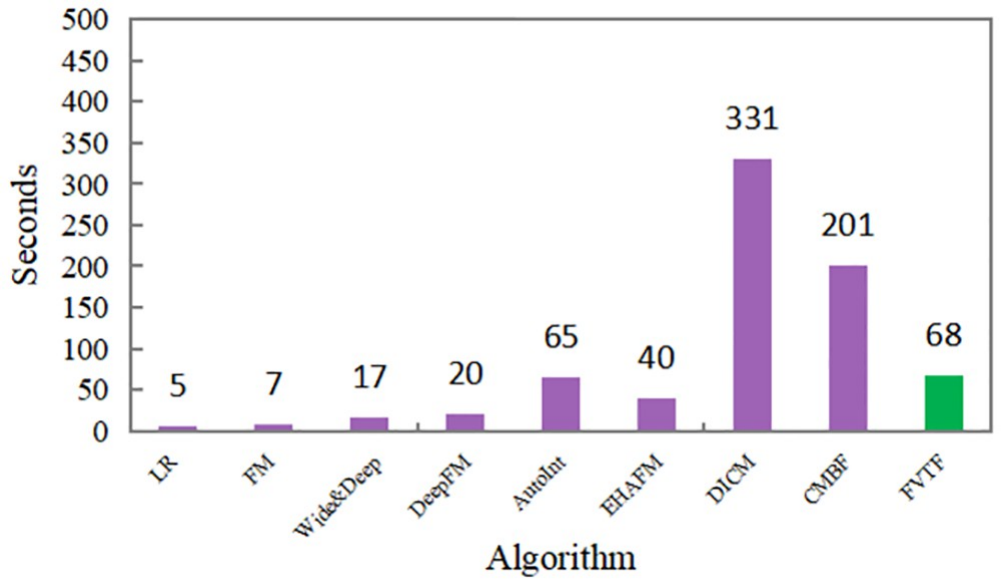
Run time per epoch.

We compared FVTF with other recommendation algorithms in terms of efficiency, comparing the training time of each epoch, and the experimental results are shown in Fig 4. Compared with most single-modal algorithms, FVTF takes a longer time to train an epoch, but such algorithms ignore image features and have poorer prediction performance. However, compared with other deep learning recommendation algorithms that fuse multi-modal features, our algorithm takes much less time to train an epoch than them and achieves optimal performance. This is due to the low complexity of the feature fusion and interaction module we designed, and the fact that separating user features from item features for feature interaction reduces non-essential computations.

#### Ablation experiments

We conducted ablation experiments on FVTF on Movielens-1M to verify the effectiveness of the feature fusion and interaction method designed in our paper and analyzed the effects of the number of feature interaction layer layers, feature fusion dimension, and the number of attention heads on the model performance.

As shown in Table 4, we can see that compared with the Multihead-Self-Attention and MLP methods, our designed feature fusion and interaction method achieves better performance, with 1.4% improvement in AUC and 4.5% reduction in Logloss compared with Multihead-Self-Attention, which verifies the This validates the effectiveness of the method. And we can see that the performance of the model improves as the number of layers increases when the feature interaction layer is ≤4, but decreases when the feature interaction layer is >4. This may be because the combination of features above the 5th order has less information useful for prediction and tends to lead to overfitting.

**Table 4:** Performance comparison of multimodal feature fusion approaches.

Fusion Method		AUC	Logloss
*MLP*		0.8802	0.3354
*Multihead* − *Self* − *Attention*		0.8821	0.3326
*OurApproach*(*layers*)	1	0.8878	0.3261
	2	0.8916	0.3221
	3	0.8936	0.3201
	4	**0.8945**	**0.3176**
	5	0.8940	0.3205

**Fig 5:**
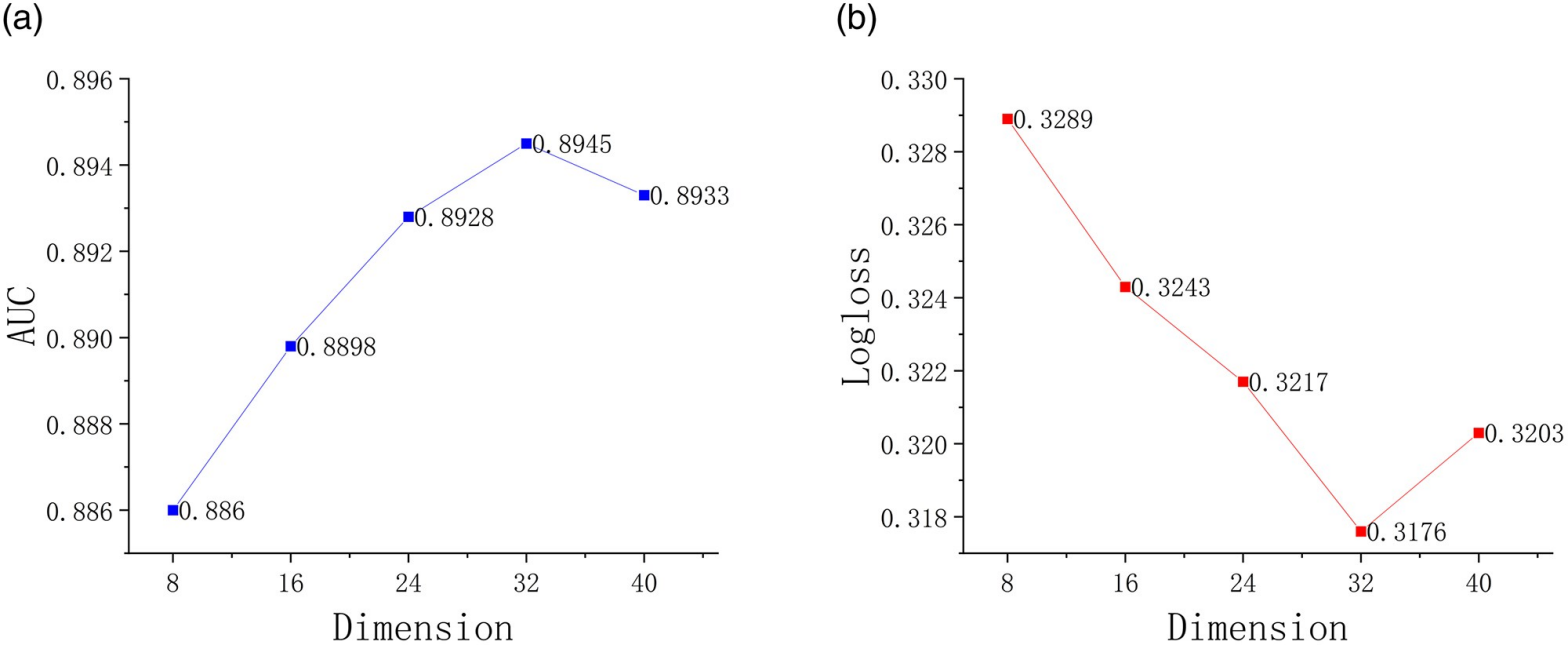
Influence of feature fusion dimension on algorithm performance. (a) AUC. (b) Logloss.

As shown in Fig 5, the model performance is improved as the feature fusion dimension increases, and the model effect tends to be flat when the feature fusion dimension is ≥32. This is because when the feature fusion dimension increases, the higher-order representation of features can accommodate more information of other features, making the fused representation of features more expressive.

**Fig 6:**
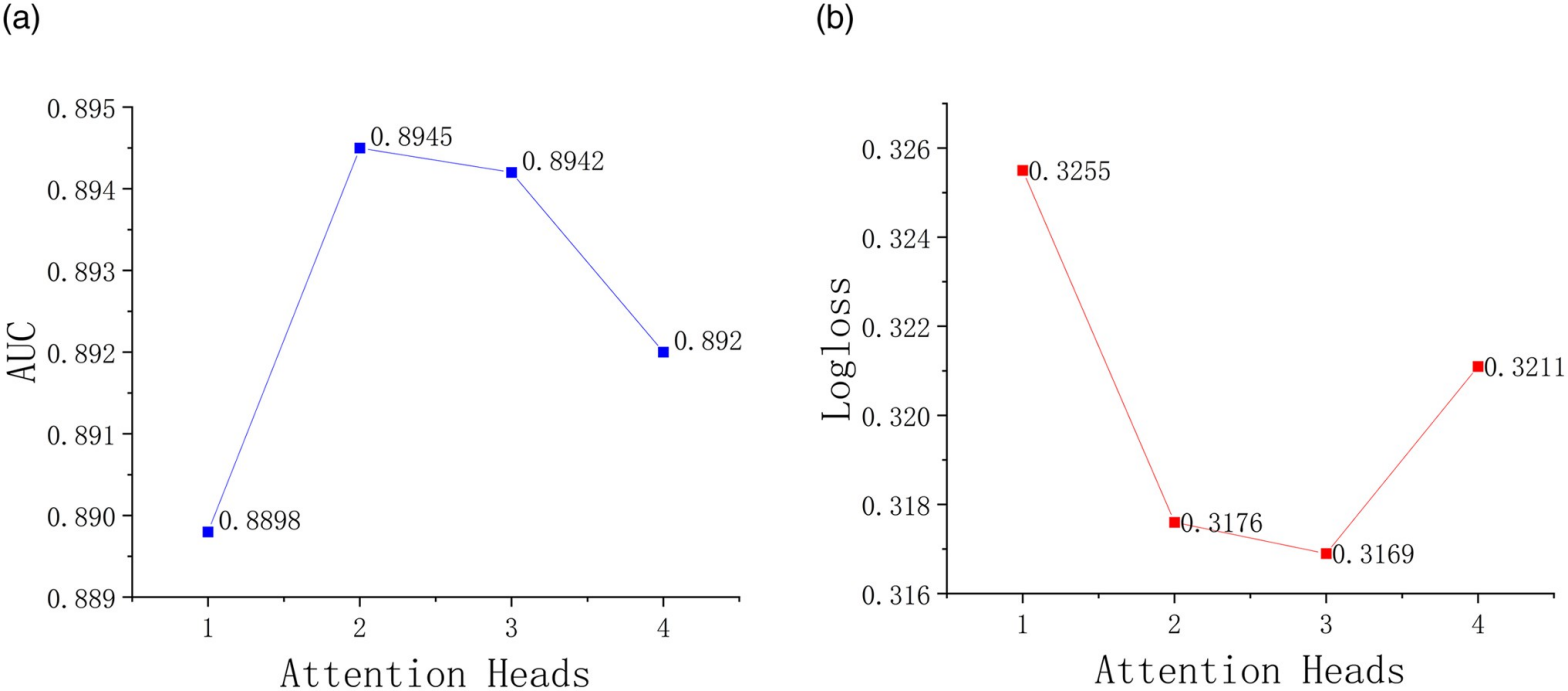
Influence of feature fusion dimension on algorithm performance. (a) AUC. (b) Logloss.

As shown in Fig 6, we compare the performance of the model at different numbers of attention heads. We can see that when the number of attention heads is 2, there is a significant improvement compared to a single attention head. The reason is that using multiple attention heads can integrate information from multiple subspaces and improve the generalization ability of the model. However, when the number of attention heads is ≥3, the AUC and Logloss tend to be flat, and even suffer from overfitting problems, which affects the final effect of the model.

## Conclusion

In this paper, we propose a multi-modal recommendation algorithm (FVTF) that fuses visual features with textual features. FVTF extracts valuable visual information from user history behavior sequences and target items as a complement of textual features to alleviate data sparsity and cold start problems in recommender systems. The results of our experiments on the Movielens-1M dataset show that FVTF has optimal performance and high efficiency compared to benchmark algorithms. In addition, the results of ablation experiments show that our approach is much better than MLP and Multi-head Self-Attention.

In the future, there are two main considerations: (1) since FVTF only utilizes information from two modalities (image and text), we will consider adding features from more modalities (e.g., sound, video, etc.); (2) since FVTF only considers a few historical click behaviors closest to the time of the target item, we will consider modeling long- and short-term visual preferences to further improve the algorithm performance.
